# Application of Cluster Analysis Technology in Visualization Research of Movie Review Data

**DOI:** 10.1155/2022/7756896

**Published:** 2022-07-16

**Authors:** Bin Xu, Cheng Chen, Jong-Hoon Yang

**Affiliations:** Department of Digital Image in Sangmyung University, Seoul 03015, Republic of Korea

## Abstract

In order to improve the reference value of film review data, this paper combines clustering analysis technology to construct a film review clustering visualization research system to improve the visualization effect of film reviews. Moreover, this paper analyzes online reviews based on the method of type-2 fuzzy sets and determines the interval of type-2 fuzzy sets of each factor set of commodity status in commercial stores by considering both the language and specific numbers of keywords in online review texts. In addition, this paper analyzes intelligent control through practical application and sets the expected target of the control object. Finally, according to the fuzzy rules and word calculation, this paper determines the interference or improvement measures that can be referenced to form a closed-loop control. The experimental research shows that cluster analysis technology can play a certain role in the visual analysis of film review data.

## 1. Introduction

With the rapid development of the Internet, film review data has become more and more open and diverse, and it has attracted more and more scientists' attention. In particular, on film websites, unconstrained users freely express their personal opinions and comments, which also forms a certain cultural phenomenon [1]. Moreover, film review data tend to be more appreciative and promotional, and film review data also tend to be different and fluid. Due to the popularity and prosperity of online media, the analysis of film reviews has been pushed to a climax [2], and it is far from enough to analyze film review data through human perception of film reviews. Therefore, more and more leaders in the computer industry conduct data analysis by processing and converting data, and making the data clearer and clearer through visualization, and the analysis results are deeply rooted in the hearts of the people [3].

By using visualization technology to visualize film review data, researchers break the “black box” of traditional analysis data [4], which can greatly enhance users' trust in analysis results. Visualization technology application methods mainly include the following aspects. First, in the data preprocessing stage, the data are directly reflected in the form of graphics to provide users with an intuitive impression, which can not only help users to understand the data from a macro perspective [5] but also help users determine the direction of analyzing the data. Second, in the data analysis stage, after the film review data are subjected to word segmentation, word frequency extraction, and other operations, these words can be displayed in a visual way. Moreover, it intuitively allows users to judge the graphics, deepens the user's understanding of the data, and enhances the interaction between data analysis and human knowledge, so it is a perfect fusion of data analysis and visualization technology. Third, in the result display stage, the analysis results of film review data are used to generate understandable graphs using visualization techniques [6]. In addition, the analysis process can also be displayed in a graphic image, which allows users to have a deep and intuitive understanding, and also makes the data analysis results clearer.

A good movie usually has many elements, such as a reasonable plot, superb acting, grand scenes, and shocking sound effects. These influencing factors are also frequently discussed in film reviews (especially long-form reviews). In order to enable our system to better understand the content of long film reviews, and more accurately identify which aspect of the film a certain long film review text discusses. We need to establish a relatively complete set of movie review keywords, such as the three keywords of plot, story, and events should correspond to the element of “plot” [7]; and the three keywords of sound effects, sound, and music should correspond to “sound” element. Although the work of establishing a keyword list for film reviews can be achieved manually, because Chinese has a large number of vocabulary, it is tantamount to looking for a needle in a haystack. In addition to the richness and complexity of Chinese expressions, it is difficult for us to manually find all the corresponding elements in keywords. Therefore, we need to use computer technology to help retrieve massive vocabulary and find these target keywords [8].

The class is to divide a data set into different classes or clusters according to a certain standard (such as a distance criterion, that is, the distance between data points) so that the similarity of data objects in the same cluster is as large as possible, and at the same time they are not in the same cluster. The data objects in a cluster are also as diverse as possible. We can specifically understand that after clustering, the data of the same class should be clustered together as much as possible and the data of different classes should be separated as much as possible [9]. The research of cluster analysis mainly focuses on distance-based clustering, fuzzy relation-based clustering, and objective function-based clustering. In the field of data mining, clustering methods include statistical methods, machine learning methods, and database-oriented methods [10].

The keywords of text are the smallest units used to understand the text. By extracting keywords that are closely related to the theme and connotation of the text, the meaning of the text expression can be grasped more quickly. In this paper, a machine learning algorithm is used for text processing of large-scale movie review text data. First, keyword extraction technology is performed, and then the movie review data are visually analyzed, so as to deeply and clearly describe the theme of the review and the user's emotional tendency [11]. The beginning of the research and application of text keyword extraction technology is marked by the automatic document tagging method based on word frequency statistics proposed by IBM Corporation of the United States. Since then, it has attracted the attention and exploration of many domestic and foreign researchers and formed several major categories, such as machine learning analysis methods, statistical analysis methods, and network analysis methods [3].

The keyword extraction process can be regarded as a binary classification problem to determine whether a word is a keyword, and the requirement is to establish a classification model based on features. Commonly used models are decision tree (DT), Naive Bayes (NB), support vector machine (SVM), hidden Markov model (HMM), conditional random field (CRF) model, etc. Turneyt proposed the Genex model, which uses a decision tree algorithm as a classifier and uses word frequency and parts of speech as features. Frankt proposed the KEA model, which implements the function of automatically extracting keywords using a Naive Bayes classifier [12]. These two models together become the benchmark system for supervised method models for automatic keyword extraction. The algorithm model of supervised learning often needs the support of a stable large-scale corpus during training, which is a large amount of data materials stored in the computer that are real and effective and have specific annotations [13] using statistical analysis methods to extract keywords. It is simpler to say and easier to understand. Theoretically, no training data and knowledge base are needed, and a collection of keywords can be obtained by processing information such as word frequency statistics, part-of-speech filtering, mean, and variance, through artificially set rules. There are three commonly used statistical and quantitative indicators for keyword judgment, namely, word-vector-based statistical and quantitative indicators; word-based document location statistical and quantitative indicators; and word-based associated information statistical and quantitative indicators.

Online reviews are a form of word-of-mouth. The storability and ease of processing of word of mouth information make word-of-mouth spread longer. Due to the increasing influence of word-of-mouth on consumers in the online environment, many scholars have begun to explore the impact of word-of-mouth on performance at the macro-group level. [14]. Relevant research can be summarized as three levels of research: the impact of word-of-mouth on corporate operating income, the impact of word-of-mouth on the promotion of new movies, and the impact of word-of-mouth on corporate stock value. However, the specifics that then affect the company's revenue, the promotion of the movie, and the value of the stock are all based on the analysis of word-of-mouth, the language of paper reviews. The influencing factors of the content of online comment information include the comprehensive evaluation of the event itself and are also affected by factors such as self-orientation, community orientation, business orientation, and reciprocity motivation [15]. It has been mentioned in the research on the effectiveness of online reviews that its effectiveness includes four dimensions, information content, information sources, movie types, and publication time. Therefore, in order to analyze the content of online reviews, it is necessary to discuss many factors and dynamic dimensions of online review information content [16].

This paper combines the cluster analysis technology to construct a film review cluster visualization research system, improve the film review visualization effect, promote the film to be better understood by people, and make it easier for people to choose excellent films to watch.

## 2. Comprehensive Evaluation and Language Dynamics Analysis of Film Reviews under IT2FS

### 2.1. Analysis of Film Review Language Based on Type 2 Fuzzy Sets

**Figure 1:**
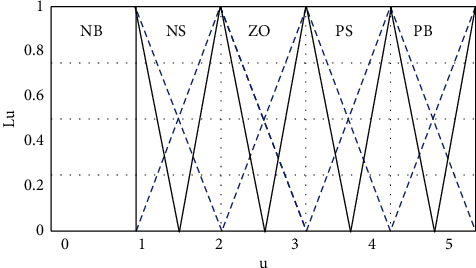
FOU of the base word membership function.

This paper discusses an analysis of online reviews based on the same type of film. According to the practice process, and survey statistics and cluster analysis are calculated. We assume that Ω_*n*_={*u*_*n*1_, *u*_*n*2_,…, *u*_*nm*_} is a discrete time-varying universe, where *u*_*ni*_(i=1,2,…) represents the same film in the *i*th theater in the *n*th film evaluation. On the domain of discourse Ω_*n*_, we assume that the five base words NB, NS, ZO, PS, and PB represent the *n*th satisfaction evaluation “very dissatisfied,” “dissatisfied,” “average,” “satisfied,” and “very satisfied,” respectively, the corresponding fuzzy sets are *ω*_1_^*n*^`*ω*_2_^*n*^`*ω*_3_^*n*^`*ω*_4_^*n*^, and *ω*_5_^*n*^, respectively. *ω*_*k*_^*n*^(*u*_*ni*_) represents the degree to which the scores of similar films in the *i*th theater in the *n*th satisfaction evaluation belong to the fuzzy set *ω*_*k*_^*n*^(*k*=1,2,3,4,5), as shown in Figure 1.

After applying the division, its membership function can be expressed as

(1) $$\begin{array}{c} \omega_{jk}^{n}=\int_{u\in \Omega }\int_{x\in L_{ujk}^{n}}\frac{\mu_{\omega_{jk}^{n}}\left(u,x\right)}{\left(u,x\right)},L_{ujk}^{n}\subseteq \left[0,1\right]\text{and }\mu_{\omega_{jk}^{n}}\left(u,x\right)=1. \end{array}$$

Among them, there are

(2) $$\begin{array}{c} L_{ujk}^{n}=\left[\underline{{\text{g}}_{jk}^{n}},\bar{{\text{g}}_{jk}^{n}}\right],\\ \text{FOU}\left(\omega_{jk}^{n}\right)=\underset{u\subset \Omega }{\cup }u\times L_{ujk}^{n}. \end{array}$$

Among them, $L_{ujk}^{n}=\left[{\text{g}}_{jk}^{n}\bar{{\text{g}}_{jk}^{n}}\right]$, the lower boundary ${\text{g}}_{jk}^{n}\bar{{\text{g}}_{jk}^{n}}$ is based on FOUK as shown in the following equation, and its functions can be expressed as follows:

(3) $$\begin{array}{c} \underline{{\text{g}}_{j1}^{n}}=\left\{\begin{array}{cc} 1 & 0\leq z_{ji}^{n}<1\\ \frac{1.5-z_{ji}^{n}}{0.5} & 1\leq z_{ji}^{n}<1.5,{\text{g}}_{j2}^{n}\\ 0 & 1.5\leq z_{ji}^{n}\leq 5 \end{array}=\left\{\begin{array}{cc} 0, & 0\leq z_{ji}^{n}\leq 1.5\\ \frac{z_{ji}^{n}-1.5}{0.5}, & 1.5<z_{ji}^{n}\leq 2\\ \frac{2.5-z_{ji}^{n}}{0.5}, & 2<z_{ji}^{n}\leq 2.5\\ 0, & 2.5<z_{ji}^{n}\leq 5 \end{array},\right.\right.\\ \underline{{\text{g}}_{j3}^{n}}=\left\{\begin{array}{cc} 0, & 0\leq z_{ji}^{n}\leq 2.5\\ \frac{z_{ji}^{n}-2.5}{0.5}, & 2.5<z_{ji}^{n}\leq 3\\ \frac{3.5-z_{ji}^{n}}{0.5}, & 3<z_{ji}^{n}\leq 3.5\\ 0, & 3.5<z_{ji}^{n}\leq 5 \end{array}\;\underline{{\text{g}}_{j4}^{n}}=\left\{\begin{array}{cc} 0, & 0\leq z_{ji}^{n}\leq 3.5\\ \frac{z_{ji}^{n}-3.5}{0.5}, & 3.5<z_{ji}^{n}\leq 4\\ \frac{4.5-z_{ji}^{n}}{0.5}, & 4<z_{ji}^{n}\leq 4.5\\ 0, & 4.5<z_{ji}^{n}\leq 5 \end{array},\right.\right.\\ \underline{{\text{g}}_{j5}^{n}}=\left\{\begin{array}{cc} 0, & 0\leq z_{ji}^{n}\leq 4.5\\ \frac{z_{ji}^{n}-4.5}{0.5}, & 4.5<z_{ji}^{n}\leq {}^{5^{{}^{\circ}}} \end{array}\right.. \end{array}$$

According to the FOU of the following, the upper boundary $\bar{{\text{g}}_{jk}^{n}}$ can be expressed as

(4) $$\begin{array}{c} \bar{{\text{g}}_{j1}^{n}}=\left\{\begin{array}{cc} 1 & 0\leq z_{ji}^{n}<1\\ 2-z_{ji}^{n} & 1\leq z_{ji}^{n}<2\\ 0 & 2\leq z_{ji}^{n}\leq 5 \end{array}\;,\bar{{\text{g}}_{j2}^{n}}=\left\{\begin{array}{cc} 0, & 0\leq z_{ji}^{n}\leq 1\\ z_{ji}^{n}-1, & 1<z_{ji}^{n}\leq 2\\ 3-z_{ji}^{n}, & 2<z_{ji}^{n}\leq 3\\ 0, & 3<z_{ji}^{n}\leq 5 \end{array},\right.\right.\\ \bar{{\text{g}}_{j3}^{n}}=\left\{\begin{array}{cc} 0, & 0\leq z_{ji}^{n}\leq 2\\ z_{ji}^{n}-2, & 2<z_{ji}^{n}\leq 3\\ 4-z_{ji}^{n}, & 3<z_{ji}^{n}\leq 4\\ 0, & 3.5<z_{ji}^{n}\leq 5 \end{array},\bar{{\text{g}}_{j4}^{n}}=\left\{\begin{array}{cc} 0, & 0\leq z_{ji}^{n}\leq 3\\ z_{ji}^{n}-3, & 3<z_{ji}^{n}\leq 4\\ 5-z_{ji}^{n}, & 4<z_{ji}^{n}\leq 5 \end{array}\right.\right.,\\ \bar{{\text{g}}_{j5}^{n}}=\left\{\begin{array}{cc} 0, & 0\leq z_{ji}^{n}\leq 4\\ z_{ji}^{n}-4, & 4<z_{ji}^{n}\leq 5 \end{array}\right., \end{array}$$

where *z*_*ji*_^*n*^ is determined by the total number of online comments, the number of negative comments, the number of moderate comments, the number of general comments, the number of positive comments, and the number of very positive comments. Its calculation method is as follows.

#### 2.1.1. Text Keywords Are Language Words

The domain of discourse performs part-of-speech tagging and word frequency statistics on the comment language in the *t*_*n*_ time period, and extracts text keywords based on the results of word frequency statistics. We assume that there are a total of *N*_*t*_*n*__ comments in the *t*_*n*_ time period, a total of *N*_*t*_*n*_1_ comments in the middle, and a total of *N*_*t*_*t*_2_ comments in the negative comments. Keywords are counted. Through the cluster analysis method in Section 3, it can be obtained that the descriptions of each influencing factor are *a*_*t*_*n*_/1_(*j*=1,2,3,…) items for “bad review,” *a*_*t*_*k*_/2_ items for “moderate review,” *a*_*t*,*j*4_ items for “good review,” and *a*_*t*_*n*_*j*5_ items for “very good review.” According to the actual data statistics and analysis results, the semantic scale of the comment sets are named “bad comment,” “moderate comment,” “general comment,” “good comment,” and “excellent,” respectively.” Assigned as *p*_*t*_*k*_/1_′, *p*_*t*_*n*_/2_′, *p*_*t*_*n*_*β*3_′, , *p*_*t*_*k*_,4_′, *p*_*t*_*n*_*j*5_′ accordingly.

The composition weight vector is as follows:

(5) $$\begin{array}{c} P_{t_{n}j}^{\prime }=\left(p_{t_{n}j1}^{\prime },p_{t_{n}/2}^{\prime },p_{t_{n}\beta 3}^{\prime },p_{t_{nj},}^{\prime },p_{t_{n}j5}^{\prime }\right). \end{array}$$

Using the weighted average fuzzy set operator to synthesize the weight vector *P*_*t*_*n*_*j*_′ and the online review evaluation matrix *R*_*t*_*n*_,*i*_′, we can get

(6) $$\begin{array}{c} Z_{i}^{n}=P_{t_{n}j}^{\prime }•R_{t_{n}i}^{\prime }=\left(z_{1i}^{n},z_{2i}^{n},z_{3i}^{n},\ldots ,z_{ji}^{n},\ldots \right). \end{array}$$

Among them, there are

(7) $$\begin{array}{c} R_{t_{n}i}^{\prime }=\left(\begin{array}{ccccc} a_{t_{n}11} & a_{t_{n}21} & \ldots & a_{t_{n}j1} & \ldots \\ a_{t_{n}12} & a_{t_{n}22} & \ldots & a_{t_{n}j2} & \ldots \\ a_{t_{n}13} & a_{t_{n}23} & \ldots & a_{t_{n}j3} & \ldots \\ a_{t_{n}j14} & a_{t_{n}24} & \ldots & a_{t_{n}j4} & \ldots \\ a_{t_{n}15} & a_{t_{n}25} & \ldots & a_{t_{n}j5} & \ldots \end{array}\right),\\ z_{ji}^{n}=\left(1/N_{t_{n}}\right)•\left(p_{t_{n}j1}^{\prime },p_{t_{n}j2}^{\prime },p_{t_{n}j3}^{\prime },p_{t_{n}j4}^{\prime },p_{t_{n}j5}^{\prime }\right)\left(\begin{array}{c} a_{t_{n}j1}\\ a_{t_{n}j2}\\ a_{t_{n}j3}\\ a_{t_{n}j4}\\ a_{t_{n}j5} \end{array}\right). \end{array}$$

Among them, the total number of comments is *N*_*t*_*n*__, the number of medium comments is *N*_*t*_*n*_1_, and the number of bad comments is *N*_*t*_*n*_2_.

It has the following relationship with the number of “bad reviews” *a*_*t*_*k*_/1_(*j*=1,2,3,…), the number of “moderate reviews” *a*_*t*_*s*/2__, the number of “general reviews” *a*_*t*_*t*/3__, the number of “good reviews” *a*_*t*_*n*_/4_, and the number of “very good reviews” *a*_*t*_*h*_*j*5_ obtained by the cluster analysis method

(8) $$\begin{array}{c} a_{t_{n}j3}=N_{t_{n}1}+N_{t_{n}2}-a_{t_{n}1}-a_{t_{n2}}\\ a_{t_{n}j4}=N_{t_{n}}-\left(N_{t_{n}1}+N_{t_{n}2}\right)-a_{t_{n}j5}. \end{array}$$

#### 2.1.2. The Text Keyword Is a Numerical Value

In the actual situation, the keywords of some factors are specific numerical values. For example, the expression form of the speed of ticket issuance and the speed of ticket issuance in online comments generally emphasize the speed at a specific time, which is based on the expression form of the comment language. Taking this as an example, we might as well set the keyword (that is, the specific value in the comment) of the ticket issuance time and ticket issuance speed in the *t*_*n*_ time period as *c*_*t*_*n*__′, and the expected value as *c*_*t*_*n*_′′_′′. The following relationship exists:

(9) $$\begin{array}{c} c_{t_{n}j}^{\prime }\in \left[0,d_{t_{h}j}\right)\text{ and }c_{t_{n}j}^{\prime }\geq \frac{c_{t_{n}j}^{\prime \prime }}{2},\\ \frac{2d_{t_{5}j}}{5}\leq 2c_{t_{n}j}^{\prime \prime }\leq d_{t_{nj}}. \end{array}$$

Then, there is

(10) $$\begin{array}{c} z_{ji}^{n}=5\left(0.75-\frac{c_{t_{n}j}^{\prime }-c_{t_{n}j}^{\prime \prime }}{d_{t_{n}j}}\right). \end{array}$$

### 2.2. Comprehensive Evaluation of Film Reviews

The paper adopts the fuzzy comprehensive evaluation method. It mainly evaluates similar movies in different theaters through online review data sources. The specific steps are as follows:

(1) Setting the factor set
Through the keyword clustering analysis method (see Section 3), *α*=0.75 is taken, and the factor set of the content described by the keyword, that is, the content described by the keyword, is {*s*_1_, *s*_2_, *s*_3_, *s*_4_}. It represents the “service attitude,” “ticket issuance speed,” “ticket issuance speed,” and “film quality” of the film theater, respectively.
(2) Determining the evaluation set
In the *n*th film evaluation, regarding the factor s_*j*_(*j*=1,2,3,4), the evaluation set *V*={NB, NS, ZO, PS,PB} of the film represents the *n*th satisfaction evaluation “very dissatisfied,” “dissatisfied,” “average,” “satisfied,” and “very satisfied.” Its corresponding fuzzy set membership function set *V*′={*ω*_*j*1_^*n*^, *ω*_*j*2_^*n*^, *ω*_*j*3_^*n*^, *ω*_*j*4_^*n*^, *ω*_*j*5_^*n*^}.
(3) Establishing a weight set
The weight set Q={0.25, 0.2, 0.15, 0.4} is set.
(4) Obtaining the judgment matrixSimilar films in different theaters are evaluated by analyzing online review data. That is, similar films *u*_*ni*_ in each film theater on Ω_*n*_ are evaluated. According to the factor s_*j*_(*j*=1,2,3,4) in the factor set, the online comment keywords are analyzed, and then the membership degree *r*_*ni*_^*jk*^ of the fuzzy set *z*_*ni*_^*j*^ on the evaluation set *ω*_*jk*_^*n*^ is calculated by *X*.We assume that $\underline{r_{ni}^{j}}=\left(\begin{array}{ccccc} \underline{r_{ni}^{j1}} & \underline{r_{ni}^{j2}} & \underline{r_{ni}^{j3}} & \underline{r_{ni}^{j4}} & \underline{r_{ni}^{j5}} \end{array}\right)$, then the judgment matrix *R*_*ni*_ can be expressed as
  (11) $$\begin{array}{c} R_{ni}=\left\{\begin{array}{ccccc} r_{ni}^{11} & r_{ni}^{12} & r_{ni}^{13} & r_{ni}^{14} & r_{ni}^{15}\\ r_{ni}^{21} & r_{ni}^{22} & r_{ni}^{23} & r_{ni}^{24} & r_{ni}^{25}\\ r_{ni}^{31} & r_{ni}^{32} & r_{ni}^{33} & r_{ni}^{34} & r_{ni}^{35}\\ r_{ni}^{41} & r_{ni}^{42} & r_{ni}^{43} & r_{ni}^{44} & r_{ni}^{45} \end{array}\right\}. \end{array}$$
We assume that $\bar{r_{ni}^{j}}=\left(\begin{array}{ccccc} \bar{r_{ni}^{ji}} & \bar{r_{ni}^{j2}} & \bar{r_{ni}^{j3}} & \bar{r_{ni}^{j4}} & \bar{r_{ni}^{j5}} \end{array}\right)$, then the judgment matrix $\bar{R_{ni}}$ can be expressed as
  (12) $$\begin{array}{c} \bar{R_{ni}}=\left\{\begin{array}{ccccc} \bar{r_{ni}^{11}} & \bar{r_{ni}^{12}} & \bar{r_{ni}^{13}} & \bar{r_{ni}^{14}} & \bar{r_{ni}^{15}}\\ \bar{r_{ni}^{21}} & \bar{r_{ni}^{22}} & \bar{r_{ni}^{23}} & \bar{r_{ni}^{24}} & \bar{r_{ni}^{25}}\\ \bar{r_{ni}^{31}} & \bar{r_{ni}^{32}} & \bar{r_{ni}^{33}} & \bar{r_{ni}^{33}} & \bar{r_{ni}^{35}}\\ \bar{r_{ni}^{41}} & \bar{r_{ni}^{42}} & \bar{r_{ni}^{43}} & \bar{r_{ni}^{44}} & \bar{r_{ni}^{45}} \end{array}\right\}. \end{array}$$
(5) Comprehensive evaluation.

We assume that *s*=*s*_1_ ⊗ *s*_2_ ⊗ *s*_3_ ⊗ *s*_4_ means that the four factor sets are integrated.

Moreover, the *n*th comprehensive evaluation conclusions “bad review,” “moderate review,” “general review,” “good review,” and “excellent review” are expressed as *C*_1_^*n*^, *C*_2_^*n*^, *C*_3_^*n*^, *C*_4_^*n*^, *C*_5_^*n*^, respectively. *C*_*k*_^*n*^ and *ω*_*jk*_^*n*^ have the same membership function; here, *k* = 1,2,3,4,5.

We assume $B_{ni}=Q^{{}^{\circ}}R_{ni}=\left(\begin{array}{ccccc} b_{ni}^{1} & b_{ni}^{2} & b_{ni}^{3} & b_{ni}^{4} & b_{ni}^{5} \end{array}\right)$, that is,

(13) $$\begin{array}{c} \underline{B_{ni}}=Q^{{}^{\circ}}\underline{R_{ni}}=\left(\begin{array}{ccccc} b_{ni}^{1} & \underline{b_{ni}^{2}} & \underline{b_{ni}^{3}} & \underline{b_{ni}^{4}} & \underline{b_{ni}^{5}} \end{array}\right),\\ \bar{B_{ni}}=Q^{{}^{\circ}}\bar{R_{ni}}=\left(\begin{array}{ccccc} \bar{b_{ni}^{1}} & \bar{b_{ni}^{2}} & \bar{b_{ni}^{3}} & \bar{b_{ni}^{4}} & \bar{b_{ni}^{5}} \end{array}\right). \end{array}$$

Here, $b_{ni}^{k}=\left[b_{ni}^{k},\bar{b_{ni}^{k}}\right]\left(k=1,2,3,4,5\right)$ means that *u*_*ni*_ comprehensively evaluates the membership degree of *C*_*k*_^*n*^. Furthermore, there are

(14) $$\begin{array}{c} C_{k}^{n}=\sum_{i=1}^{m_{n}}\frac{1/b_{ni}^{k}}{u_{ni}}=\sum_{i=1}^{m_{n}}\frac{1/\left[b_{ni}^{k}\bar{b_{ni}^{k}}\right]}{u_{ni}}. \end{array}$$

In this way, the language dynamics trajectory of the comprehensive evaluation value of the similar film theaters is formed as follows:

(15) $$\begin{array}{c} C_{k}^{1},C_{k}^{2},\cdots ,C_{k}^{n},\cdots . \end{array}$$

### 2.3. The Linguistic Dynamics Trajectory of Film Criticism in the Time-Varying Universe

Through the analysis of online reviews, further improvement measures can be implemented for theaters to achieve closed-loop control of theater film transactions. Cinema improvement measures are to exert influence in a planned and step-by-step manner after analyzing the current situation and problems of the cinema, so as to make the operation change towards the desired goal.

In the paper, IT2FS is used to describe the cinema improvement measures. Due to the different factors that affect the current situation of the theater, the theater film is not the same as the problem now. Different factors have different degrees of influence, and the corresponding improvement measures will also be different. Through the keyword cluster analysis method, *α*=0.75 is taken. The content of the keyword description, that is, the factor set of the content described by the keyword, is {*s*_1_, *s*_2_, *s*_3_, *s*_4_}, and it represents the “service attitude,” “ticketing speed,” “ticketing speed,” and “film quality” of the film theater, respectively. Regarding the factor *s*_*j*_(*j*=1,2,3,4), the corresponding fuzzy set membership function set of the film evaluation set is {*ω*_*j*1_^*n*^, *ω*_*j*2_^*n*^, *ω*_*j*3_^*n*^, *ω*_*j*4_^*n*^, *ω*_*j*5_^*n*^}={NB, NS, ZO, PS, PB}. The corresponding improvement measures are *l*_*j*_(*l*_*j*_=4) categories, and the membership function set of the strength of each category is {*v*_1*i*_^*n*^, *v*_2*i*_^*n*^, *v*_3*i*_^*n*^}={Bad, Normal, Good}. In the actual cinema film reviews, the data collection time of online reviews is generally the sales in the last 30 days. In the paper, the length of the data collection period is 30 days. Taking time *t* as a cycle, the fuzzy rules are established through the experience of experts and the statistical survey of online actual data as follows:

 
*R*_1_: if *s*_1_(*n*) is *ω*_1*m*_*k*_1___, *U*_1_ is *v*_*jl*_1__, then *s*_1_(*n*+1) is *ω*_1*m*_81__;
 
*R*_2_: if *s*_2_(*n*) is *ω*_2*m*_*k*_2___, *U*_2_ is *v*_*jl*_*r*2__, then *S*_2_(*n*+1) is *ω*_2*m*_*g*2__;
 
*R*_3_: if *s*_3_(*n*) is *ω*_3*m*_*k*3__, *U*_2_ is *v*_*jl*_*r*3__, then *s*_3_(*n*+1) is *ω*_3*m*_83__;
 
*R*_4_: if *s*_4_(*n*) is *ω*_4*m*_*k*4__, *U*_2_ is *v*_*jl*_*r*4__, then *s*_4_(*n*+1) is *ω*_4*m*_*g*4__.

Among them, *m*_*k*_*j*__, *m*_*g*_*j*__ ∈ [1,5], *l*_*r*_*j*__ ∈ [1,3], and they are all positive integers. *R*_*j*_ is a fuzzy rule defined on Ω_*j*_, including 15 fuzzy subrules, which are defined as follows:

 
*R*_*j*_(1,1): if *s*_*J*_(*n*) is NB, *U*_*j*_ is bad, then *s*_*j*_(*n*+1) is NB;
 
*R*_*j*_(1,2): if *s*_*j*_(*n*) is NB, *U*_*j*_ is normal, then *s*_*j*_(*n*+1) is NS;
 
*R*_*j*_(1,3): if *s*_*j*_(*n*) is NB, *U*_*j*_ is good, then *s*_*j*_(*n*+1) is ZO;
 
*R*_*j*_(2,1): if *s*_*J*_(*n*) is NS, *U*_*j*_ is bad, then *s*_*j*_(*n*+1) is NS;
 
*R*_*j*_(2,2): if *s*_*j*_(*n*) is NS, *U*_*j*_ is normal, then *s*_*j*_(*n*+1) is ZO;
 
*R*_*j*_(2,3): if *s*_*J*_(*n*) is *NS*,  *U*_*j*_ is good, then *s*_*j*_(*n*+1) is PS;
 
*R*_*j*_(3,1): if *s*_*j*_(*n*) is ZO, *U*_*j*_ is bad, then *s*_*j*_(*n*+1) is ZO;
 
*R*_*j*_(3,2): if *s*_*j*_(*n*) is ZO, *U*_*j*_ is normal, then *s*_*j*_(*n*+1) is ZO;
 
*R*_*j*_(3,3): if *s*_*J*_(*n*) is ZO, *U*_*j*_ is good, then *s*_*j*_(*n*+1) is PS;
 
*R*_*j*_(4,1): if *s*_*J*_(*n*) is PS, *U*_*j*_ is bad, then *s*_*j*_(*n*+1) is PS;
 
*R*_*j*_(4,2): if *s*_*j*_(*n*) is PS, *U*_*j*_ is normal, then *s*_*j*_(*n*+1) is PS;
 
*R*_*j*_(4,3): if *s*_*J*_(*n*) is PS, *U*_*j*_ is good, then *s*_*j*_(*n*+1) is PB;
 
*R*_*j*_(5,1): if *s*_*j*_(*n*) is PB, *U*_*j*_ is bad, then *s*_*j*_(*n*+1) is PB;
 
*R*_*j*_(5,2): if *s*_*J*_(*n*) is PB, *U*_*j*_ is normal, then *s*_*j*_(*n*+1) is PB;
 
*R*_*j*_(5,3): if *s*_*J*_(*n*) is PB, *U*_*j*_ is good, then *s*_*j*_(*n*+1) is PB.

The initial word *ω*_*j*_(0) is calculated through comprehensive evaluation, and the activated fuzzy rule *R*_*j*_ is determined. The improvement goal *ω*_*j*_^*∗*^ is given, then there are

(16) $$\begin{array}{c} \lambda^{*}\left(m_{k_{j}},0\right)=\omega_{j}^{*}\wedge \omega_{jm_{gj}}. \end{array}$$

Furthermore, there are

(17) $$\begin{array}{c} \lambda^{*}\left(m_{k_{j}},0\right)=\max\left\{\lambda^{*}\left(1,0\right),\lambda^{*}\left(2,0\right)\ldots ,\lambda^{*}\left(m_{k_{j}},0\right)\right\}. \end{array}$$

According to the above formula, it can be calculated that *v*_*jl*_*j*__ is the most suitable improvement measure.

**Figure 2:**
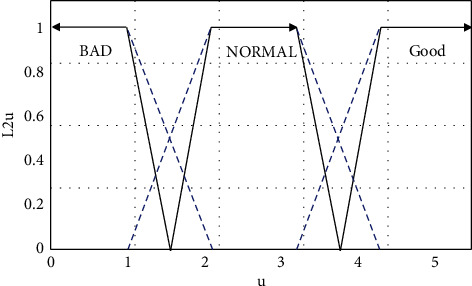
FOU of the membership function of the improvement measures.

In order to facilitate the calculation, the paper takes improvement measures from four aspects: service, ticket issuance, film source, and work efficiency. We assume that Ω_*n*_={*u*_*n*1_, *u*_*n*2_,…, *u*_*nm*_} is a discrete time-varying universe, where *u*_*ni*_(i=1,2,…). At the same time, we assume that *U*={*U*_1_, *U*_2_, *U*_3_, *U*_4_} is a discrete universe of discourse and use three fuzzy sets *v*_1*i*_^*n*^, *v*_2*i*_^*n*^, *v*_3*i*_^*n*^ to cover the universe of universe *U*, which, respectively, represent the effect of the *n*th improvement measures “poor,” “average,” and “good.” *v*_*l*_^*n*^(*u*_*ni*_)(*l*=1,2,3) represents the degree to which the evaluation scores of the corresponding improvement measures taken for the similar films in the *i*th theater belong to the fuzzy set *X*_*l*_^*n*^ after the *n*th evaluation. Corresponding to “poor,” “general,” and “good” specific measures, the FOU of its membership function is shown in Figure 2.

After applying the division, its membership function can be expressed as

(18) $$\begin{array}{c} v_{l}^{n}=\int_{u\in \Omega }\int_{x\in L_{ui}^{\prime n}}\frac{\mu_{X_{i}^{n}}\left(u,x^{\prime }\right)}{\left(u,x^{\prime }\right)},L_{ul}^{\prime n}\subseteq \left[0,1\right]\text{and}\mu_{v_{l}^{n}}\left(u,x^{\prime }\right)=1. \end{array}$$

Among them, there are

(19) $$\begin{array}{c} L_{ul}^{\prime n}=\left[\underline{{\text{g}}_{l}^{\prime n}},\bar{{\text{g}}_{l}^{\prime n}}\right],\\ FOU\left(v_{l}^{n}\right)=\underset{u\in \Omega }{\cup }u\times L_{ul}^{\prime n}. \end{array}$$

Among them, $L_{ul}^{\prime n}=\left[\underline{{\text{g}}_{l}^{\prime n}},\bar{{\text{g}}_{l}^{\prime n}}\right]$, the lower boundaries $\underline{{\text{g}}_{l}^{\prime n}},\bar{{\text{g}}_{l}^{\prime n}}$ can be expressed as

(20) $$\begin{array}{c} \underline{{\text{g}}_{1}^{\prime n}}=\left\{\begin{array}{cc} 1 & 0\leq z_{li}^{\prime n}<1\\ \frac{1.5-z_{li}^{\prime n}}{0.5} & 1\leq z_{li}^{\prime n}<1.5\\ 0 & 1.5\leq z_{li}^{\prime n}\leq 5 \end{array},\underline{{\text{g}}_{3}^{\prime n}}=\left\{\begin{array}{cc} 0 & 0\leq z_{li}^{\prime n}<3.5\\ \frac{z_{li}^{\prime n}-3.5}{0.5} & 3.5\leq z_{li}^{\prime n}<4\\ 1 & 4\leq z_{li}^{\prime n}\leq 5 \end{array}\right.\right.,\\ \underline{{\text{g}}_{2}^{\prime n}}=\left\{\begin{array}{cc} 0, & 0\leq z_{li}^{\prime n}\leq 1.5\\ \frac{z_{li}^{\prime n}-1.5}{0.5}, & 1.5<z_{li}^{\prime n}\leq 2\\ 1, & 2<z_{li}^{\prime n}\leq 3, \end{array},\bar{{\text{g}}_{2}^{\prime n}}=\left\{\begin{array}{cc} 0, & 0\leq z_{li}^{\prime n}\leq 1\\ z_{li}^{\prime n}-1, & 1<z_{li}^{\prime n}\leq 2\\ 1, & 2<z_{li}^{\prime n}\leq 3\\ \frac{3.5-z_{ji}^{n}}{0.5} & 3<z_{li}^{\prime n}\leq 3.5\\ 0, & 3.5<z_{li}^{\prime n}\leq 5 \end{array}\;\left\{\begin{array}{cc} 4-z_{ji}^{n}, & 3<z_{li}^{\prime n}\leq 4\\ 0, & 3.5<z_{li}^{\prime n}\leq 5 \end{array}\right.\right.,\right.\\ \bar{{\text{g}}_{1}^{\prime n}}=\left\{\begin{array}{cc} 1 & 0\leq z_{li}^{\prime n}<1\\ 2-z_{li}^{\prime n} & 1\leq z_{li}^{\prime n}<2\\ 0 & 2\leq z_{li}^{\prime n}\leq 5 \end{array},\bar{{\text{g}}_{3}^{\prime n}}=\left\{\begin{array}{cc} 0 & 0\leq z_{li}^{\prime n}<3\\ z_{li}^{\prime n}-3 & 3\leq z_{li}^{\prime n}<4\\ 1 & 4\leq z_{li}^{\prime n}\leq 5 \end{array}\right.\right.. \end{array}$$

Among them, *z*_*li*_^′*n*^ is the improvement intensity value indicating the corresponding improvement measures taken for the similar films in the *i*th theater after the *n*th evaluation.

### 2.4. The Language Dynamics System of IT2FS under the Time-Varying Universe

It is difficult for conventional mathematical models to study the complex system of human direct participation in film analysis. People can establish fuzzy rules based on actual experience and relevant knowledge, and then use fuzzy reasoning to analyze the system dynamically. Therefore, a language dynamics system of interval type-two fuzzy sets (IT2FSs) in the time-varying universe is proposed. We assume that *R*_1_, *R*_2_, ⋯, *R*_*n*_, ⋯ are dynamic fuzzy rules defined on the universes Ω_1_, Ω_2_, ⋯, Ω_*n*_, ⋯ and *n* is a natural number, then there are

(21) $$\begin{array}{c} R=\overset{\infty }{\underset{n=1}{\cup }}R_{n}. \end{array}$$

For the initial state word *ω*_1_ ∈ *ℜ*(Ω_1_) and the initial input word *v*_1_ ∈ *ℜ*(*U*_1_), under the action of fuzzy rules, the language dynamics trajectory is

(22) $$\begin{array}{c} \omega_{1},\omega_{2},\cdots ,\omega_{n},\cdots . \end{array}$$

Among them, there are

(23) $$\begin{array}{c} \omega_{n+1}=R\left(\omega_{n},v_{n}\right)=R^{n}\left(\omega_{1},v_{1}\right),\\ \omega_{n}\in ℜ\left(\Omega_{n}\right),v_{n}\in ℜ\left(U_{n}\right),\\ R^{n}=R_{n}{}^{\circ}R_{n-1}{}^{\circ}\cdots^{{}^{\circ}}R_{1}. \end{array}$$

The corresponding language dynamics trajectory can also be in the following form:

(24) $$\begin{array}{c} \omega_{1},R^{1}\left(\omega_{1},v_{1}\right),\cdots ,R^{n}\left(\omega_{1},v_{1}\right),\cdots . \end{array}$$

For complex systems such as film analysis, the object is generally a single individual. However, individual characteristics factor papers are available in a continuous IT2FS study. Based on the continuous IT2FS, the calculation steps of the language dynamics trajectory of IT2FS in the time-varying universe are as follows:

1. The domain of discourse of the target object is determined. According to the specific research object, the universe of discourse Ω_*t*_, *t*=1,2,3, ⋯ at time *t* is determined.
2. We assume Ω_*t*_={*u*_1_, *u*_2_, ⋯, *u*_*n*_}. If Ω_*t*_ is an incremental discrete time-varying universe, then Ω_*t*+1_={*u*_1_, *u*_2_, ⋯, *u*_*n*_, *u*_*n*+1_ ⋯ *u*_*n*+*i*_}. If Ω_*t*_ is a decreasing discrete time-varying universe, then Ω_*t*+1_={*u*_1_, *u*_2_, ⋯, *u*_*n*−*i*−1_, *u*_*n*−*i*_}. If Ω_*t*_ is a wave-type discrete time-varying universe, then Ω_*t*+1_={*a*_1_, *a*_2_, ⋯, *a*_*n*−*i*−1_, *a*_*n*−*i*_, *a*_*n*+1_ ⋯ *a*_*n*+*i*_}, *i* ≥ 1.
3. The feature set of each element in the universe of discourse is determined. According to the characteristics of the domain element, the possible characteristics of the element are determined and covered with the set base words.
4. The algorithm evaluates the object, and the paper adopts the fuzzy comprehensive evaluation. Elements in different domains of discourse at different times have different feature sets, and the same element has different membership degrees for different features at different times. Therefore, with the change of time *t*, the language dynamics trajectory of the system can be obtained.

However, in intelligent control, the author needs to set the expected target according to the control object, and then formulate interference or improvement measures. A closed-loop control is formed to obtain the expected language dynamics trajectory and complete the control task.

## 3. Research on Visualization of Film Review Data Based on Cluster Analysis Technology

**Figure 3:**
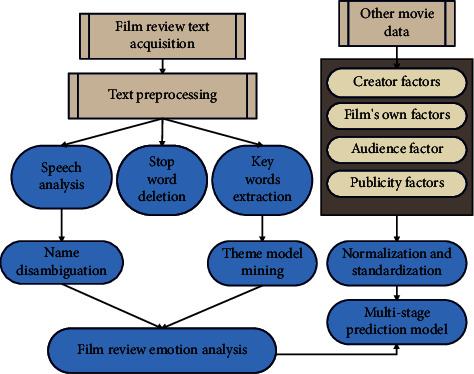
Overall framework of multistage box office forecasting.

The contribution value of an actor can be evaluated by using Weibo data, star fan data, and published content related to the film release cycle. Sentiment features are given by the analysis of film reviews. Usually, it is necessary to identify the film review for a certain main creator from a large number of comments, then perform statistical analysis on the positive and negative emotions of the film review, and combine the syntactic analysis and fan data to give the influence of the main creator of the change.  Figure 3 shows the overall framework of multistage box office prediction.

**Figure 4:**
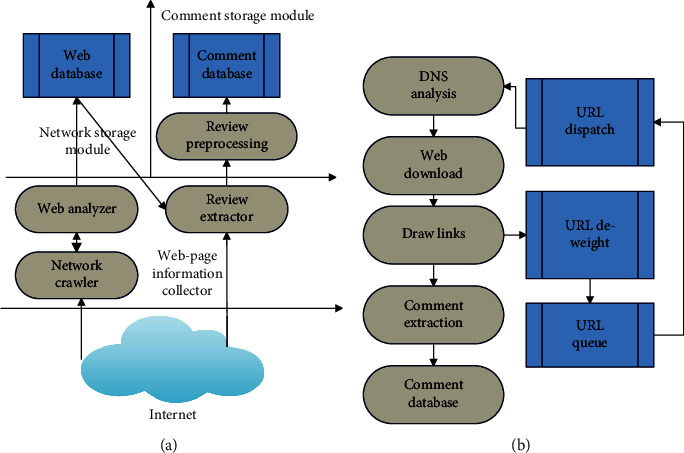
Crawler framework of film review data: (a) architecture of the review crawler and (b) flowchart of the review crawler.

The main modules of the web comment crawler are web page information collection module, web page storage module, and comment storage module, as shown in Figure 4(a). The crawler crawls the web page through the seed URL, extracts the qualified URLs in the web page, and adds them to the URL queue to prepare for crawling in the next cycle. Then, it extracts the network comments in the web page through the web page information collection module. This paper uses multithreading to implement web crawler, realizes URL deduplication through bloom filter, and crawls needed comment information. The specific execution steps are as follows, and the flowchart is shown in Figure 4(b).

**Figure 5:**
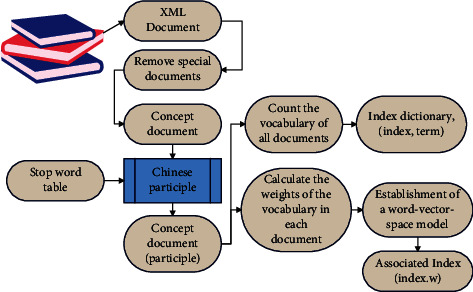
The establishment of the vector space model of words.

This paper represents a vocabulary with a set of network natural concepts, encoding reviews using a large amount of highly advanced human knowledge, which is defined by people themselves and can be easily interpreted. Through continuous development, film reviews have grown in breadth and depth over time. The establishment of the vector space model of words is shown in Figure 5. This method makes up for the problem of low accuracy in calculating similarity caused by short vocabulary and high dimensionality and few values.

**Figure 6:**
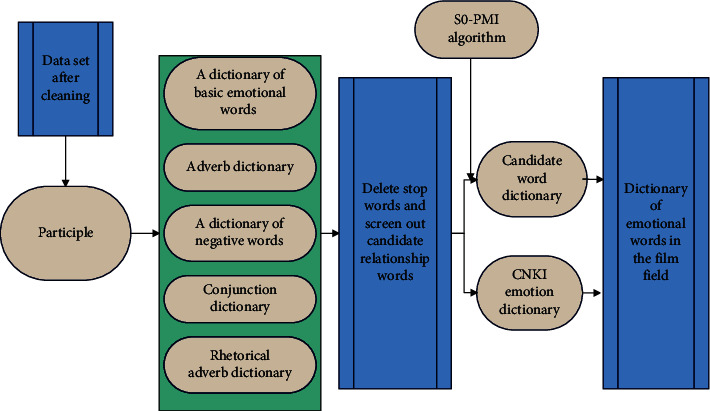
Construction framework of emotional dictionary in the film field.

Emotional dictionaries are generally not perfect. Moreover, when dealing with different text sentiment analysis tasks, the coverage of sentiment words is obviously not enough. Analyzing text information in the film industry requires building a sentiment dictionary that fits the product, and analyzing the film industry also requires building a sentiment dictionary suitable for film review analysis. Therefore, according to the actual research situation, it is necessary to mine new emotional words and add them to the emotional dictionary used for research. The process framework of constructing emotion dictionary in the film field is shown in Figure 6.

**Figure 7:**
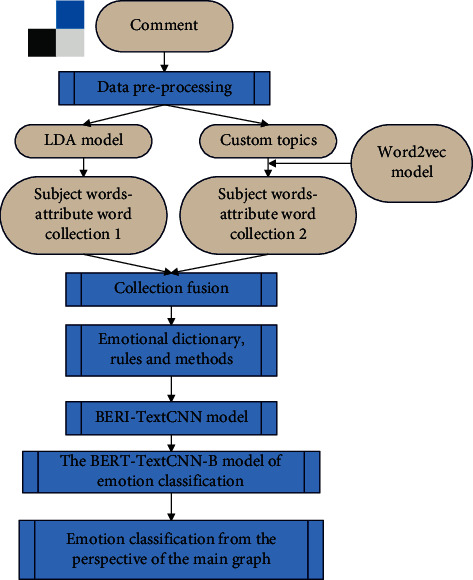
A fine-grained sentiment analysis framework for film reviews.

After data preprocessing, the LDA topic model is used for topic modeling. In order to analyze the sentiment tendency from a topic that is more in line with the market perspective, through the custom topic and the initialized attribute word, the distance between the Word2Vec word vectors is used, and the attributes with a correlation greater than 0.8 are selected for topic classification. These two subject-attribute word sets are fused to construct a subject-attribute word set, as shown in Figure 7.

**Figure 8:**
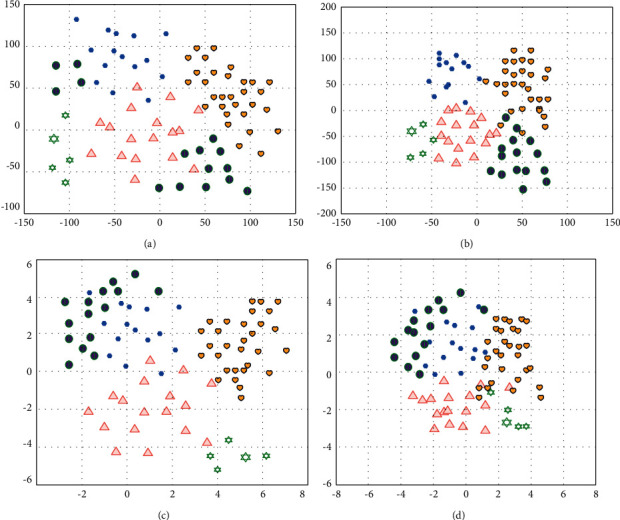
Film review clustering effect: (a) *k*-means clustering results; (b) *k*-means clustering results after introducing PCA predimension reduction; (c)clustering results of the chasing clustering algorithm without PCA prereduction; (d)clustering results of the chasing clustering algorithm after introducing PCA predimension reduction.

When reducing the dimension of film reviews, the vector dimension obtained after training for each film is 400 dimensions, which is too high. Therefore, when the traditional t-SNE algorithm is used for dimensionality reduction, the result shown in Figure 8(a) is obtained. There are problems that the clustering results are not clear, they do not meet the clustering principle of “high cohesion and low coupling,” and the significant effect between the clustering results is not ideal.

The PCA predimension reduction process is introduced, and the 400-dimensional high-dimensional vector is first reduced to 50 dimensions, and then the t-SNE dimensionality reduction algorithm is used to solve the problem of poor cluster visualization. The clustering results after introducing PCA predimension reduction are shown in Figure 8(b).

The t-SNE method with the introduction of PCA predimensionality reduction is used to visualize the results of the chasing clustering algorithm for dimensionality reduction. Comparing Figures 8(c) and 8(d), we can see that the clustering results in Figure 8(d) have better significant effect and meet the clustering requirements of “high cohesion and low coupling.”

It can be seen from the above research that cluster analysis technology can play a certain role in the visualization analysis of film review data.

## 4. Conclusion

Limited by the bottleneck of natural language understanding technology, the industry's use of film review data is still very limited. At present, no film platform or research paper can effectively mine the hidden value in film review data. The film review is the most direct evaluation of the film by the audience, and it can reflect the audience's viewing experience in the most real and detailed way. Moreover, mining the value of film review data is of great significance for us to fully understand the audience experience and scientifically guide the development and progress of the film industry. This paper combines the clustering analysis technology to construct a research system of film review cluster visualization, which can improve the visualization effect of film reviews and promote the film to be better understood by people. The experimental research shows that the cluster analysis technology can play a certain role in the visual analysis of film review data.

## Data Availability

The labeled dataset used to support the findings of this study are available from the corresponding author upon request.
